# The effect of social distance measures on COVID-19 epidemics in Europe: an interrupted time series analysis

**DOI:** 10.1007/s11357-020-00205-0

**Published:** 2020-06-11

**Authors:** Zoltán Vokó, János György Pitter

**Affiliations:** 1 Center for Health Technology Assessment, Semmelweis University, Budapest, Hungary; 2 Syreon Research Institute, Budapest, Hungary

**Keywords:** COVID-19, Interrupted time series analysis, Google community mobility reports, Social distance, Europe

## Abstract

Following the introduction of unprecedented “stay-at-home” national policies, the COVID-19 pandemic recently started declining in Europe. Our research aims were to characterize the changepoint in the flow of the COVID-19 epidemic in each European country and to evaluate the association of the level of social distancing with the observed decline in the national epidemics. Interrupted time series analyses were conducted in 28 European countries. Social distance index was calculated based on Google Community Mobility Reports. Changepoints were estimated by threshold regression, national findings were analyzed by Poisson regression, and the effect of social distancing in mixed effects Poisson regression model. Our findings identified the most probable changepoints in 28 European countries. Before changepoint, incidence of new COVID-19 cases grew by 24% per day on average. From the changepoint, this growth rate was reduced to 0.9%, 0.3% increase, and to 0.7% and 1.7% decrease by increasing social distancing quartiles. The beneficial effect of higher social distance quartiles (i.e., turning the increase into decline) was statistically significant for the fourth quartile. Notably, many countries in lower quartiles also achieved a flat epidemic curve. In these countries, other plausible COVID-19 containment measures could contribute to controlling the first wave of the disease. The association of social distance quartiles with viral spread could also be hindered by local bottlenecks in infection control. Our results allow for moderate optimism related to the gradual lifting of social distance measures in the general population, and call for specific attention to the protection of focal micro-societies enriching high-risk elderly subjects, including nursing homes and chronic care facilities.

## Introduction

After a million confirmed and 100,000 fatal European cases, the COVID-19 pandemic started declining in Europe in April 2020 (European Centre for Disease Prevention and Control 2020). This much awaited decline was headed by the introduction of unprecedented “stay-at-home” national policies in most countries, including border closure, public gathering bans, school and workplace closure, and temporary restrictions on free internal movements of the citizens (European Centre for Disease Prevention and Control 2020). These economically and socially disruptive control measures are not sustainable on the longer term (Petersen et al. 2020), and gradual restart of economy and social life is now on the political agenda throughout Europe (European Centre for Disease Prevention and Control 2020; European Commission 2020). A European roadmap to lifting the coronavirus containment measures has been framed, proposing a gradual, slow approach backed with adequate monitoring and healthcare capacity to ensure sufficient control of potential flare-ups (European Commission 2020). Planning the consecutive steps is supported by general provisions and considerations of the roadmap; however, ultimately, it remains a trial-and-error-based process due to the high uncertainty in possible consequences of any change in containment measures. Importantly, all national epidemic containment measures were introduced within a narrow time period in most countries (European Centre for Disease Prevention and Control 2020; Hale et al. 2020a). Hence, the contribution of unique interventions to the overall impact on COVID-19 spread is hard to estimate retrospectively (Imai et al. 2020; Imperial College COVID-19 Response Team Report 13 2020). As an overall measure of policy response intensity, the Blavatnik School of Government proposed a composite Stringency Index, integrating rigor and scope of multiple containment and closure policies (school and workplace closure, restrictions on gathering, international and internal movements, public transport, cancelation of public events, and information campaigns) into a single numeric parameter in the 0–100 range (Hale et al. 2020a). The same team organizes global data collection on all included indicators, as well as on economic responses and adaptations of public health systems, providing a freely available but very precious tool to overview and visualize global policy efforts. However, this Stringency Index has important limitations when tested as an explanatory factor of SARS-CoV-2 infection spread. First, the adopted categories of policy rigor and scope may aggregate heterogeneous policies (as illustrated by an amendment on 28 April 2020—see the details at (Hale et al. 2020b)). More importantly, the Stringency Index is based on sterile policy decisions, while the compliance of the population with the corresponding restrictions may vary across countries and over time. The Google COVID-19 Community Mobility Reports provide an alternative option to derive a composite stringency measure of multiple containment and closure policies (Google 2020). These community mobility reports provide daily, country-level (and sub-regional) aggregated anonymized data on time spent at different categories of places such as retail and recreation, groceries and pharmacies, parks, transit stations, workplaces, and residential areas, compared with a baseline period before the epidemic. Telemonitored mobility trends are dynamic in time and reflect real-world changes in social behavior, making them promising explanatory factors in SARS-CoV-2 infection spread control analyses.

Recently published COVID-19 microsimulation models based on social network data in the UK and USA revealed that epidemic suppression would require a complex intervention package including social distancing of the entire population, home isolation of cases, and household quarantine of their family members, supplemented with school closure, in intermittent periods adjusted to epidemic intensity and unoccupied critical care capacity (Imperial College COVID-19 Response Team Report 9 2020; Kucharski et al. 2020). However, adaptation of these microsimulation models to other countries would require rich and solid input data on local social networks. A semi-mechanistic Bayesian hierarchical model of social distancing interventions across 11 European countries was also reported, calculating daily infections from observed death rates (Imperial College COVID-19 Response Team Report 13 2020). The authors inferred that the combined application of five intervention types (lockdown, public events ban, school closure, self-isolation, and social distancing) could prevent about 59,000 COVID-19 deaths in the investigated 11 countries until the end of March 2020. Nonetheless, individual contributions of the five intervention types to the overall effect showed high uncertainty, probably because many interventions occurred on the same day or within days of each other. Important limitations of this study were the assumptions on identical effect of interventions across countries and over time, and the possible over-representation of countries with more advanced epidemics (Imperial College COVID-19 Response Team Report 13 2020). Additional reassurance whether the COVID-19 transmission was truly slowing has been warranted.

To estimate the effect of social distancing on the time trend data of the epidemic, interrupted time series analysis is an alternative approach (Aminikhanghahi and Diane 2017). This analysis can be extended to a broader range of European countries without need for sophisticated local input data collection and assumptions on between-country similarities; hence, it can broaden our current understanding of the epidemic and its association with changes in population social distance patterns. Our research aims were to identify the date when the COVID-19 pandemic started declining in each European country and to evaluate the association of the level of community mobility restrictions (social distancing) with the observed extent of decline in the national epidemics. Establishing an association of telemonitored population mobility patterns with a decline in COVID-19 spread may support policymakers in assessing the benefits of previously implemented stay-at-home policies, and in planning the gradual lifting of current restrictions.

## Methods

### Disease incidence data

Daily incidence of new COVID-19 cases by countries was obtained from the open-access database of the European Centre for Disease Prevention and Control (European Centre for Disease Prevention and Control Data 2020). Data from European Union member states and the European Free Trade Association countries were included to focus our analyses on countries with similar sociocultural characteristics and reliable estimates of changes in daily COVID-19 incidence. Data from Cyprus, Iceland, and Liechtenstein were dropped, due to the lack or scarcity of related Google community mobility reports. Data from Latvia have also been dropped, because it covered only 3 days in the observation period (see below). Accordingly, the analyses included data from 28 countries: Austria, Belgium, Bulgaria, Croatia, Czech Republic, Denmark, Estonia, Finland, France, Germany, Greece, Hungary, Ireland, Italy, Lithuania, Luxembourg, Malta, Netherlands, Norway, Poland, Portugal, Romania, Slovakia, Slovenia, Spain, Sweden, Switzerland, and the UK. Within the study period of 1 February to 18 April, the first day of observation was defined in each country as the last day when the number of the new cases was at least 5 following 2 days with less than 5 new cases. Due to missing data for some calendar days, the start of observation period was postponed to 12 March in Finland and to 16 March in Luxembourg (Table 2).

### Changepoint detection and characterization by country

**Fig. 1:**
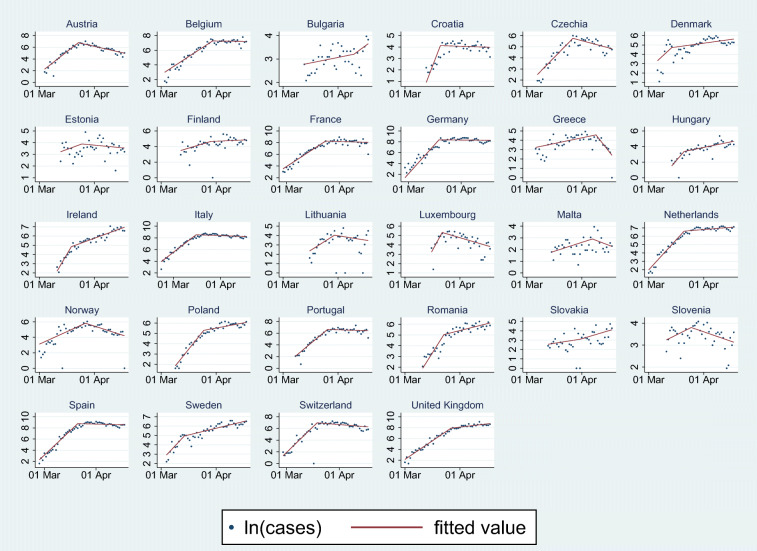
Changepoint detection and Poisson regression findings in the 28 investigated countries. Note the country-specific vertical scales

The most likely changepoint date was determined in each country by linear threshold regression models of the logarithm of daily cases over time, replacing zero daily cases in these analyses by one, and looking for threshold in the 20–80 percentile range of the country time series, using the threshold application of the statistical package STATA 16.0 (StataCorp 2019). The threshold regression using the logarithm of cases in linear regression with the usual Gaussian, homoscedastic and independent errors is a correct method to identify the threshold, but when the extent of change is estimated the count nature of the data needs to be taken into account as the non-Gaussian errors might give incorrect standard errors of the regression coefficients. Therefore the extent of change at the most probable changepoint in the reported daily incidence was estimated by country via Poisson regression models using Poisson application of STATA 16.0. Independent variables in the models were time from start of observation and time from the estimated changepoint. As the observations by country were not independent, Huber/White/sandwich variance estimator was used to estimate confidence intervals. Results of changepoint identification and Poisson regression are summarized in Table 1 and Fig. 1.

**Table 1:** First day of study period and time series analysis findings by country

Country	First day of the observation period	Changepoint date	Change of the slope at the changepoint (95% CI)	*P* value
Austria	Mar 4	Mar 24	− 0.31 [− 0.35; − 0.27]	< 0.001
Belgium	Mar 3	Mar 31	− 0.16 [− 0.20; − 0.12]	< 0.001
Bulgaria	Mar 13	Apr 11	+ 0.041 [− 0.025; 0.11]	0.22
Croatia	Mar 13	Mar 22	− 0.41 [− 0.55; − 0.27]	< 0.001
Czech Republic	Mar 7	Mar 28	− 0.21 [− 0.24; − 0.17]	< 0.001
Denmark	Mar 6	Mar 15	− 0.15 [− 0.29, − 0.0078]	0.04
Estonia	Mar 13	Mar 26	− 0.070 [− 0.17; 0.032]	0.18
Finland	Mar 12	Mar 30	− 0.059 [− 0.13; 0.014]	0.11
France	Feb 28	Mar 25	− 0.20 [− 0.24; − 0.16]	< 0.001
Germany	Feb 28	Mar 20	− 0.35 [− 0.42; − 0.28]	< 0.001
Greece	Mar 6	Apr 10	− 0.28 [− 0.36; − 0.20]	< 0.001
Hungary	Mar 14	Mar 22	− 0.22 [− 0.33; − 0.10]	< 0.001
Ireland	Mar 11	Mar 20	− 0.26 [− 0.33, − 0.19]	< 0.001
Italy	Feb 22	Mar 17	− 0.21 [− 0.23; − 0.18]	< 0.001
Lithuania	Mar 16	Mar 31	− 0.15 [− 0.26; − 0.040]	0.007
Luxembourg	Mar 16	Mar 23	− 0.40 [− 0.54; − 0.26]	< 0.001
Malta	Mar 15	Apr 8	− 0.11 [− 0.22; 0.0030]	0.057
Netherlands	Mar 1	Mar 22	− 0.21 [− 0.25; − 0.18]	< 0.001
Norway	Mar 1	Mar 28	− 0.18 [− 0.22; − 0.14]	< 0.001
Poland	Mar 9	Mar 26	− 0.18 [− 0.22; − 0.15]	< 0.001
Portugal	Mar 8	Mar 27	− 0.26 [− 0.31; − 0.22]	< 0.001
Romania	Mar 11	Mar 24	− 0.22 [− 0.29; − 0.15]	< 0.001
Slovakia	Mar 13	Apr 1	0.026 [− 0.057; 0.11]	0.54
Slovenia	Mar 11	Mar 26	− 0.068 [− 0.11; − 0.028]	0.001
Spain	Feb 27	Mar 22	− 0.29 [− 0.34; − 0.24]	< 0.001
Sweden	Mar 4	Mar 15	− 0.16 [−0.23; −0.083]	< 0.001
Switzerland	Feb 28	Mar 20	− 0.31 [− 0.36; − 0.25]	< 0.001
UK	Mar 1	Mar 28	− 0.18 [− 0.21; − 0.15]	< 0.001

### Social distance index calculation based on Google Community Mobility Reports

**Fig. 2:**
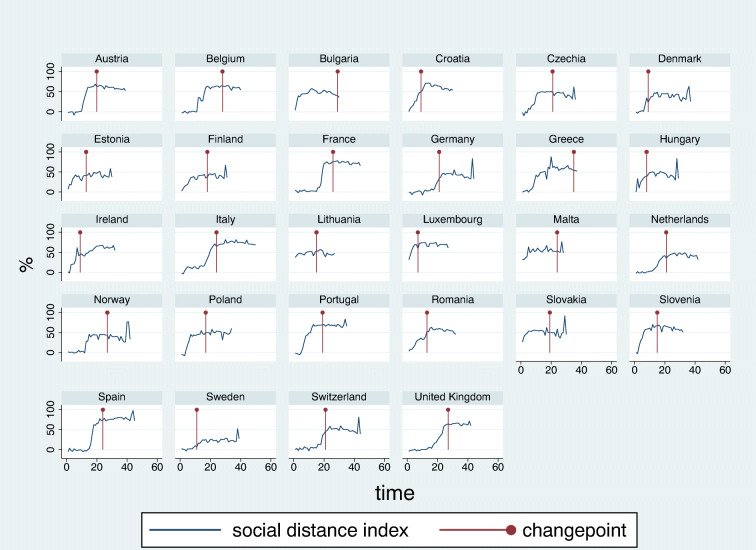
Country-specific social distance index data based on Google Community Mobility Reports. The vertical lines represent most likely changepoints in the increase of daily incidence rates

The Google COVID-19 Community Mobility Reports provide daily, country-level (and sub-regional) aggregated anonymized data on time spent at six different categories of places, compared with a baseline period before the epidemic and controlled for the weekday effect (Google 2020). In the investigated countries, largest reported decline in staying in retail and recreation, grocery and pharmacy, parks, transit stations, and workplace areas were − 96%, − 92%, − 91%, − 92%, and − 90%, respectively, while highest reported change in staying in residential areas was + 46%. These dimensions of community mobility were integrated into a social distance index. First, data on staying in parks was omitted, since its implications on social distance were considered ambiguous: staying in parks may reflect either individual or social activity. As a next step, daily change from baseline in each mobility report dimension was normalized between baseline and international maximum (see above). Finally, the normalized values were averaged, yielding a country-specific daily social distance index with a baseline of 0 and a theoretical maximum of 100. Country-specific social distance index data over time are shown in Fig. 2. For multivariate regression analysis, the average social distance index was estimated for a 14-day incubation period ending at the changepoint for each country, separately, and countries were grouped by four quartiles of this parameter (Table 2).

**Table 2:** Countries by average social distance index quartiles in a 14-day incubation period ending at the changepoint

SDI quartile	Countries by increasing social distance index	SDI	
		Range	Mean (SD)
1	Sweden, Germany, Denmark, Switzerland, Netherlands, Romania, Hungary	4.1–28.2	16.5 (8.3)
2	Ireland, Croatia, Estonia, UK, Finland, Italy, Norway	28.4–42.6	35.7 (5.1)
3	Poland, Spain, Austria, Czech Republic, Bulgaria, Lithuania, France	43.3–49.0	45.7 (2.2)
4	Slovakia, Slovenia, Malta, Luxembourg, Greece, Portugal, Belgium	53.6–61.3	57.2 (2.7)

*SDI* social distance index, *SD* standard deviation

### Effect of social distancing on the spread of the epidemic

Daily new cases were modeled via mixed effects Poisson regression with gamma random effect (Sutradhar and Jowaheer 2003) in the xtpoisson application of STATA 16.0, using countries as random effect. Fixed effects in the model were time from the start of observation period, time from changepoint, and the interaction between the latter and the quartiles of the average social distance index in 14 days ending at changepoint, reflecting an incubation period of 1–14 days before diagnosis of new cases (European Centre for Disease Prevention and Control 2020).

### Statistical software and code

All analyses were conducted in STATA 16.0 (StataCorp 2019), and double-checked in R (R Core Team R 2017) using packages chngpt (Fong et al. 2017) and hglm (Rönnegård et al. 2010; Alam et al. 2015).

## Results

Most likely changepoints and the estimated extent of change are summarized in Table 2 and depicted in Fig. 1. The identified changepoints were associated with statistically significant alteration in daily COVID-19 incidence in 23 of the 28 investigated countries, and all significant findings exhibited a decline in epidemic spread. Findings of the multinational regression analysis are summarized in Table 3. Translating the model coefficients into incidence rate ratios shows that before changepoint, incidence of new COVID-19 cases grew by 24% per day (IRR 1.24) on average. From the changepoint, this growth rate reduced to 0.9%, 0.3% increase, and to 0.7% and 1.7% decrease by increasing SDI quartiles. The beneficial effect of higher social distance quartiles (i.e., turning the increase into decline) was statistically significant for the fourth quartile.

**Table 3:** Effect of social distance measure on the rate of the spread of the epidemic

Variable	Rate ratio* (95% CI)	*P* value
Time from start till changepoint	1.238 (1.196–1.281)	< 10^−3^
Time from changepoint in SDI		
1st quartile	1.009 (0.998–1.020)	0.12
2nd quartile	1.003 (0.979–1.028)	0.82
3rd quartile	0.993 (0.985–1.000)	0.054
4th quartile	0.983 (0.971–0.995)	0.007

*CI* confidence interval, *SDI* social distance index

^*^Corresponding to 1-day difference in time

## Discussion

Our analysis identified the most probable changepoint in the flow of the COVID-19 epidemic in 28 European countries and found a clear dose-response association of the observed flattening of the epidemic curve with increasing social distance index derived from Google Community Mobility Reports. Countries in the highest SDI quartile achieved a statistically significant decline of the epidemic, with less and less new cases every day, while countries with the least stringent SDI increase also greatly reduced the initially high growth rate of incident COVID-19 cases. Accordingly, it can be inferred that the unprecedented “stay-at-home” national policies meaningfully contributed to the suppression of the COVID-19 pandemic in Europe. Countries which achieved on average only 16% of the maximum observed level of the decrease in social contacts showed already a large reduction in the spread of the epidemic. On the other hand, restrictions on internal movements of the citizens are obviously not the only contributors to this decline: contact tracing and isolation, widescale use of individual protective equipment, keeping safe interpersonal distance in public places, and proper hand hygiene are all plausible contributors to stopping the first wave of the pandemic in Europe (European Commission 2020; Imperial College COVID-19 Response Team Report 9 2020).

Notably, the importance of local micro-epidemic chains in the overall COVID-19 epidemic is better and better recognized. Nursing homes are known to be predisposed to having high transmission rates for infectious diseases for many reasons including crowding, sharing bathroom facilities, social contacts, and low preparedness for infection control. Unfortunately, COVID-19 does not seem to be an exception in this respect (Davidson and Szanton 2020; Trabucchi and De Leo 2020). According to a WHO report on 23 April, up to half of those who have died from COVID-19 in the European Region were resident in long-term care facilities (World Health Organization Statement 2020). To prevent COVID-19 transmission in nursing homes and other chronic care facilities enriching high-risk elderly patient groups, effective local infection control measures are clearly more relevant than general interventions targeting the country population as a whole, without specific focus on critical hot spots of the epidemic. Such a discrepancy between global and local containment measures may also explain the relatively small difference in the slowing of the epidemic by different level of social distancing. Therefore, in parallel with the gradual lifting of country-level COVID-19 spread control measures, special attention must be paid to ensure adequate local infection control in nursing homes and chronic inpatient care facilities, in compliance with the European roadmap to lifting coronavirus containment measures (European Commission 2020) and the corresponding recommendations of the Centers for Disease control and Prevention (Centers for Disease Control and Prevention Key Strategies 2020).

## Conclusions

The unprecedented “stay-at-home” national policies meaningfully contributed to the suppression of the COVID-19 pandemic in Europe, which can be detected in macro level time trend analysis. However, the importance of several other interventions introduced in parallel must be noted as well, and our findings could be shaped also by the important distinction between country-level and institution-level preparedness. Our findings allow for moderate optimism related to the gradual lifting of social distance measures in the general population, and call for specific attention to the protection of focal micro-societies enriching high-risk elderly subjects, including nursing homes and chronic care facilities.

## Data Availability

Publicly available data was used for the analysis (see in the Acknowledgments).
